# Tailoring Atomoxetine Release Rate from DLP 3D-Printed Tablets Using Artificial Neural Networks: Influence of Tablet Thickness and Drug Loading

**DOI:** 10.3390/molecules26010111

**Published:** 2020-12-29

**Authors:** Gordana Stanojević, Djordje Medarević, Ivana Adamov, Nikola Pešić, Jovana Kovačević, Svetlana Ibrić

**Affiliations:** 1 Institute for Medicines and Medical Devices of Montenegro, Ivana Crnojevića 64a, 81000 Podgorica, Montenegro; gordana.boljevic@cinmed.me; 2 Department of Pharmaceutical Technology and Cosmetology, Faculty of Pharmacy, University of Belgrade, Vojvode Stepe 450, 11221 Belgrade, Serbia; djordje.medarevic@pharmacy.bg.ac.rs (D.M.); ivana.adamov@pharmacy.bg.ac.rs (I.A.); nikola.pesic@pharmacy.bg.ac.rs (N.P.); Jovana.Kovacevic@hemofarm.com (J.K.)

**Keywords:** three-dimensional (3D) printing, additive manufacturing, digital light processing (DLP), personalized therapy, neural networks, optimization, release rate

## Abstract

Various three-dimensional printing (3DP) technologies have been investigated so far in relation to their potential to produce customizable medicines and medical devices. The aim of this study was to examine the possibility of tailoring drug release rates from immediate to prolonged release by varying the tablet thickness and the drug loading, as well as to develop artificial neural network (ANN) predictive models for atomoxetine (ATH) release rate from DLP 3D-printed tablets. Photoreactive mixtures were comprised of poly(ethylene glycol) diacrylate (PEGDA) and poly(ethylene glycol) 400 in a constant ratio of 3:1, water, photoinitiator and ATH as a model drug whose content was varied from 5% to 20% (*w*/*w*). Designed 3D models of cylindrical shape tablets were of constant diameter, but different thickness. A series of tablets with doses ranging from 2.06 mg to 37.48 mg, exhibiting immediate- and modified-release profiles were successfully fabricated, confirming the potential of this technology in manufacturing dosage forms on demand, with the possibility to adjust the dose and release behavior by varying drug loading and dimensions of tablets. DSC (differential scanning calorimetry), XRPD (X-ray powder diffraction) and microscopic analysis showed that ATH remained in a crystalline form in tablets, while FTIR spectroscopy confirmed that no interactions occurred between ATH and polymers.

## 1. Introduction

Three-dimensional (3D) printing, also known as additive manufacturing, is a process which enables the production of solid objects of practically any shape by digitally controlled deposition of materials in a layer-by-layer manner [1]. After approval of the first 3D-printed medicine Spritam^®^ (Aprecia Pharmaceuticals) by the United States Food and Drug Administration (FDA) in 2015 [2], this technology, used in various fields of industry, has attracted huge attention from the professional and scientific public, showing the potential to revolutionize the pharmaceutical field and the way medicines are designed and produced [3,4].

The concept of personalized medicine seeks to tailor medical treatment to the individual need of each patient based on their own characteristics, needs and state of disease, in order to achieve the most desirable therapeutic outcomes [5]. One of the most important aspects of personalized medicine is individualization and adjustment of the dosage to a single patient. Traditional large-scale manufacturing processes are not offering many possibilities to implement this concept, because medicines are usually produced in a limited number of doses, which do not exactly match the need of each patient. This is particularly an issue in the field of solid dosage forms, where splitting or crushing of tablets is the only way to adjust the dose, which can result an inadequate quantity of medicine or violation of the function of the film coating when it has the role of modifying the release rate of the active substance [6]. On the other hand, the application of 3D printing in the pharmaceutical industry could make it possible to move from a “one size fits all” approach to the manufacturing of small batches of patient-tailored medicines [7,8,9], as it allows flexible adjustment of the dose, appropriate release profiles and a combination of drugs in a single polypill [10,11,12,13,14].

Various types of printing technologies have been used so far in the manufacturing of dosage forms, including fused deposition modelling (FDM), based on material extrusion [15,16], selective laser sintering (SLS), based on the laser binding of powder particles [17,18], and stereolithography (SLA) and digital light processing (DLP), based on polymerization and solidification of a photocurable resin which contains the active substance upon light irradiation [19]. While SLA uses a laser beam to cure the layers, DLP uses a digital light projector to gradually expose and cure the whole layer of photopolymerizable resin simultaneously [20,21]. After printing each consecutive layer, the moving platform immerses into the resin tank, covering previously cured layers with photocurable resin, enabling each following layer to be cured by the light source and to adhere to the previous one, until the printing of the object is completed. Compared to other 3D-printing techniques, SLA and DLP are fast, produce objects with high printing resolution and accuracy, and also prevent drugs from thermal degradation as heating is minimized. On the other hand, the main disadvantage is that there are a limited number of photocrosslinkable polymers that can be used in preparing photoreactive mixtures, as well as the fact that they are currently not on the generally recognized as safe (GRAS) list [21].

So far, DLP printers have demonstrated their capacity to create dental models with high accuracy [22], as well as patient-specific drug delivery devices, including microneedle arrays on finger splints [23], hydrogel microneedles [24], non-dissolving suppository molds [25] and various implants [26]. Despite numerous advantages, the potential application of the DLP technique in the production and optimization of oral dosage forms has not been fully explored. Kadry et al., for the first time, assessed the feasibility of DLP 3D printers in the fabrication of solid oral dosage forms, using poly(ethylene glycol) diacrylate (PEGDA) and poly(ethylene glycol) dimethacrylate (PEGDMA) as photoreactive polymers and theophylline as a model drug [27]. After optimization of various printing parameters including UV intensity, exposure time, layer thickness and polymer concentration, various types of modified-release tablets with or without perforation were printed. Madzarevic et al. prepared 11 formulations of ibuprofen tablets following the D-optimal mixture design from Design Expert software. Two artificial neural networks (ANN) (STATISTICA 7.0 and MATLAB R2014b) were used in order to investigate how the formulation factors affect the printability, as well as to predict and optimize extended ibuprofen release from printed formulations. This study described that appropriate ANN allows understanding of the input–output relationship in DLP printing of pharmaceutics [28]. Krkobabic et al. investigated the effect of three different hydrophilic excipients on the paracetamol release rate from DLP-printed tablets, with the aim to improve the very slow and incomplete drug release from tablets produced by DLP technology. The study demonstrated that the addition of mannitol, sodium chloride and poly(ethylene glycol) 400 (PEG 400) increased paracetamol release from printed tablets, with a different effect on mechanical characteristics of tablets [29]. In another study, Krkobabic et al. demonstrated successful DLP printing with an active substance suspended in photopolymer mixture [30]. In a study by Larush et al., pH-responsive hydrogels loaded with sulforhodamine B were produced using DLP 3D printing [31]. Printed hydrogels showed high swelling and faster drug release at higher pH, which could be particularly useful for targeted drug release in the small intestine. Oral dosage forms fabricated with DLP 3D printing, providing immediate release of the active substance, have not been yet described in the literature. 

Artificial neural networks are one of the main tools used in machine learning; they are brain-inspired systems which are intended to replicate the way that humans learn. Neural networks consist of input and output layers, as well as a hidden layer (one or more), consisting of units (often called “neurons” or “nodes”) that transform the input into something that the output layer can use. There are several types of networks, which have specifics and differ in architecture and learning algorithms. Likewise, one chosen type of a network can differ in the number of layers, the number of neurons in each layer, the number of epochs used for learning, as well as the type of functions used (activation and postsynaptic function). ANNs have been used in pharmaceutical development for the last twenty years, mainly in formulation and process optimization. The advantage of ANN over statistical techniques, such as Design of Experiments (DoE) using multiple regression analysis, is the ability to simultaneously model, optimize and predict a large number of variables. Furthermore, ANNs can handle incomplete, missing data or those whose structure is not predefined [32]. 

The aim of this study was to develop ANN predictive models for atomoxetine hydrochloride (ATH) release rates from DLP 3D-printed tablets. Moreover, our aim was to examine the possibility of tailoring drug release rates from immediate to prolonged release by varying the tablet thickness and the drug loading. ATH was chosen as a model drug because it is used in the wide range of doses (500 micrograms/kg daily to 100 mg or 1.4 mg/kg daily) mostly within the child population in the treatment of attention deficit hyperactivity disorder (ADHD) [33], which makes it a suitable candidate for individualization of therapy. 

## 2. Results and Discussion

### 2.1. 3D-Printing Process

**Figure 1:**
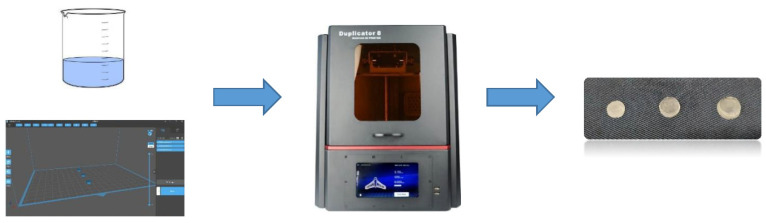
Schematic illustration of the DLP (digital light processing) printing process and obtained tablets of 1 mm, 2 mm and 3 mm thickness.

Cylindrical tablets of constant diameter but growing thickness, containing different amounts of ATH, were successfully fabricated using a DLP 3D printer (Figure 1).

The printing process was fast and efficient and lasted from 4 min 30 s to 13 min 30 s for 20 tablets of each formulation, depending on the thickness of the tablets. This confirms previously reported advantages of DLP 3D printers in terms of the ability to fabricate a bulk amount of tablets in a short period of time at room temperature [27,28,29], making it possible, together with other 3D-printing technologies, to be used within clinical settings and community pharmacies for the preparation of customized dosage forms and medical devices [34,35]. 

To date, DLP printers have been exploited to produce tablets and hydrogels containing different drugs, e.g., ATH [30], theophylline [27], ibuprofen [28], paracetamol [29] and sulforhodamine B [31], polymers/mixtures of polymers, including PEGDA, PEG 300 and PEG 400, and photoinitiators (PI) such as Irgacure 2959, riboflavin and diphenyl (2,4,6-trimethylbenzoyl) phosphine oxide (DPPO). Unlike commercially available resins, when it comes to the printing of the dosage forms, it is necessary to establish optimal printing parameters for each formulation separately, in order to ensure good adhesion of the tablets to the building platform and to avoid significant variations in tablet dimensions and consequently drug loading [14,28].

The working wavelength of the Wanhao Duplicator 8 3D printer used in this study is 405 nm. The same working wavelength was used in our previous studies, where the model drugs were ibuprofen [28] and ATH [30]. Both of the studies demonstrated that no drug degradation occurred during the 3D-printing process. The stability of the drug upon UV light irradiation is an important issue and it has to be evaluated while considering the feasibility of DLP 3D-printing technology for preparation of tablets with a particular drug substance.

### 2.2. Appearance, Weight, Dimensions and Drug Content 

**Figure 2:**
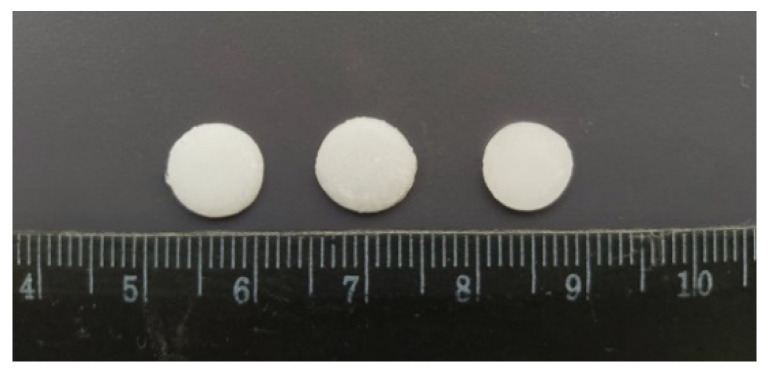
Fabricated tablets containing 15% ATH (atomoxetine hydrochloride) with diameters of 8 mm and 2 mm thickness.

All of the fabricated tablets had a smooth surface and were of uniform shape in accordance with the developed 3D model (Figure 2). Tablets were stored at ambient conditions and did not show signs of changes in consistency and shape.

The measured weight, dimensions and drug content of all formulations are shown in Table 1.

ATH is approved for use in children aged 6 years and older in the treatment of ADHD [33] and is marketed in the form of tablets and capsules within a wide range of doses: 10 mg, 18 mg, 25 mg, 40 mg, 60 mg, 80 mg and 100 mg [36]. Most of the studies that aimed to demonstrate the feasibility of various 3D-printing techniques in the fabrication of pharmaceutical dosage forms have been conducted with a clinically irrelevant amount of drugs. The influence of differences in surface area of tablets on drug-release behavior has already been investigated and described in papers published in the field of DLP 3D-printing research [27]. In the present study, by varying the thickness of the tablets and the amount of drug in the photoreactive mixture from 5% to 20%, we have managed to fabricate series of tablets with the content of ATH within its therapeutic range. This confirmed the potential application of DLP 3D-printing technology in the individualization of therapy by producing tablets with different drug loadings, which would enable adjustment of dosage to the need of each particular patient by taking tablets individually or in combination. Furthermore, the smallest amount of drug incorporated into the fabricated tablets was around 2 mg, indicating the potential for fabrication of low-dose medicines. In a study by Bracken et al. conducted among children aged 4–12, tablets with a diameter of 8 mm were rated to be the most acceptable tablet size; also, beside tablet size, as the second most important factor, if they had to take tablets every day, the participants reported taste of the tablets [37]. Taking into account that the size and taste of a tablet are fundamental to the ability and willingness of a child to swallow it, small tablets that can be fabricated with the DLP 3D printer containing a fraction of the required dose may be considered as a solution to improve both the acceptability and adequate dosing. As ATH was used as a model drug, which is mainly indicated in children for the treatment of ADHD, the diameter of the tablet was constant at 8 mm, while the thickness of the tablets varied, thus achieving different drug loadings and release profiles. Masking the unpleasant taste of a drug is another issue which can be overcome by adding taste-masking substances or natural sweeteners like mannitol, whose suitability for DLP printing has already been demonstrated by Krkobabic et al. [29].

The diameter of the fabricated tablets varied to a greater extent with respect to their thickness, especially with the increase of the amount of ATH in the formulations. This can be explained by the larger amount of suspended particles and their scattering phenomena onto the light beam [30]. Additionally, greater variation in diameter in comparison to the designed 3D model was observed for 2-mm- and 3-mm-thick tablets, which can be attributed to the longer exposure time needed for their successful printing. Therefore, it is very important to set the exposure time to the smallest value that enables solidification in order to minimize the impact on the dimensions of the tablets and consequently avoid dosage variation.

### 2.3. Mechanical Properties of Tablets

**Figure 3:**
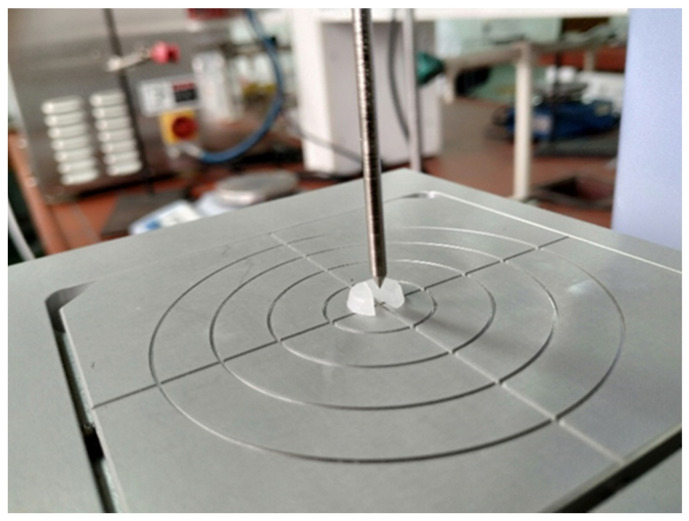
Determination of mechanical properties of the tablets on EZ-LX Compact Table-Top Testing Machine.

Since quality assessment guidelines for 3D-printed medicines have not been established yet, and some of the traditional testing methods are not entirely suitable for their control due to the differences in consistency and firmness in comparison with conventional solid dosage forms, alternative approaches have to be developed in order to examine and optimize 3D-printed tablets. Because all of the prepared formulations contained the same ratio of PEGDA and PEG 400, the content of water and PI was constant in all of them and all tablets were fabricated using the same printing parameters, a EZ-LX Compact Table-Top Testing Machine (Figure 3) was used to measure the penetration force, which reflects the mechanical properties of the tablets, in order to investigate the potential effect of the drug load on the hardness of the fabricated tablets. 

The results (Table 2) suggest that a higher content of ATH, as well as an increase in the tablet thickness, required a higher force to push the needle probe into the tablet, indicating their influence on the mechanical properties of the fabricated tablets. Taking into consideration that the hardness of the tablets may influence the drug release rate, the impact of those factors were further investigated in the in vitro dissolution testing.

### 2.4. Dissolution Profiles

**Figure 4:**
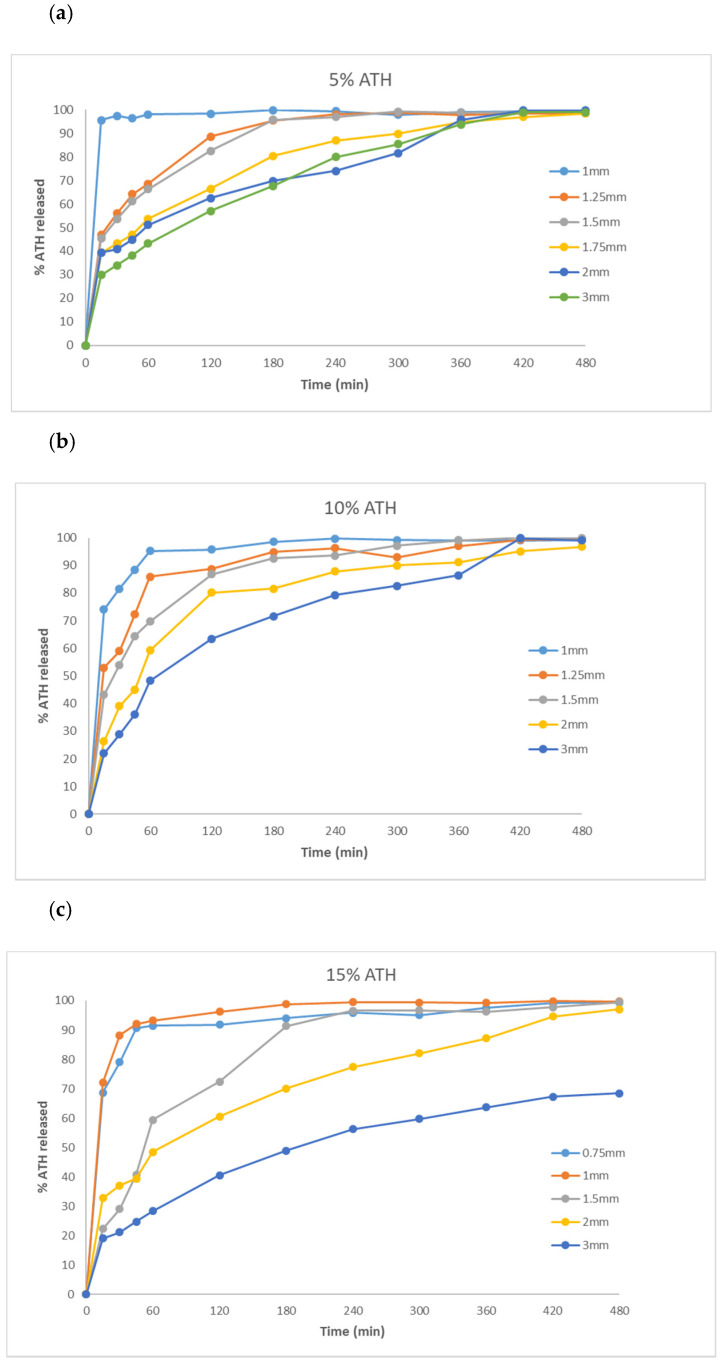

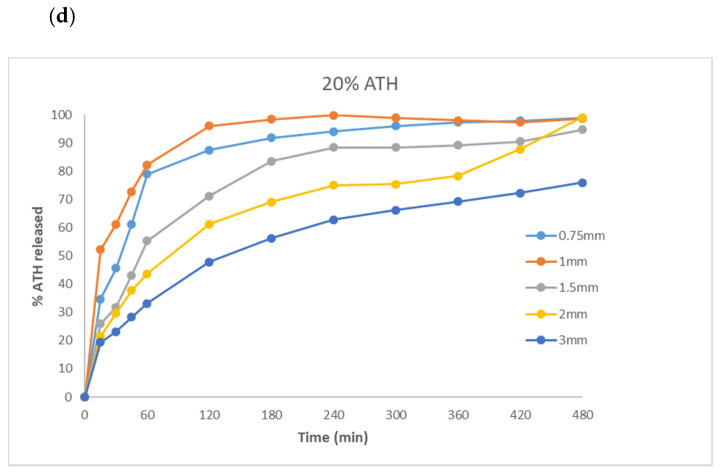
Dissolution profiles of fabricated tablets containing: (**a**) 5%; (**b**) 10%; (**c**) 15%; and (**d**) 20% of ATH.

Similarly to previously published studies [27,28], printed tablets remained intact after dissolution testing, without signs of erosion or disintegration. Dissolution profiles for all formulations are presented in Figure 4.

According to Chapter 5.17.1, Recommendation on dissolution testing, in the European Pharmacopoeia Edition 10.2, in terms of the expression of dissolution specification for conventional-release dosage forms, the acceptance criteria at level S1 are that at least 80% of the active substance is released within a specified time, typically 45 min or less. Results of the dissolution testing of fabricated tablets showed that more than 80% of ATH was released in 45 min from 1-mm-thick tablets containing 5%, 10% and 15% of ATH, and from 0.75-mm-thick tablets containing 15% of ATH. To the best of our knowledge, there are no immediate-release dosage forms fabricated by one of the vat polymerization technologies reported so far. In the first study that demonstrated the feasibility of SLA technology in the manufacturing of oral dosage forms, tablets with modified-release characteristics were obtained [21]. Wang et al. demonstrated that a higher content of PEGDA slows down the release rate, due to the higher degree of crosslinking and slower diffusion of the drug from the polymeric matrix. Other attempts to modify and improve the drug release rate include the addition of various excipients, such as water [38], sodium chloride and mannitol [29]. Besides that, the drug release rate can be controlled by changing the geometry of tablets or making a different number of perforations in order to increase the SA/V ratio [27,39,40]. The possibility to fabricate immediate-release dosage forms via DLP 3D technology is very significant because releasing drugs in a timely manner is important for optimal therapeutic effects. Besides that, different clinical conditions require different types of release profiles. There is no comparison in the literature between the standard casting method for the manufacture of immediate-release dosage forms and 3D printing on the efficiency of drug release. However, the advantage of printing is that it is possible to print films or tablets of different thicknesses very precisely and quickly.

Other fabricated tablets expressed modified-release characteristics, with complete release of ATH except from 3-mm-thick tablets containing 15% and 20% of the drug, which released 68.42% and 72.29% of drug, respectively, within 8 h. This slower release rate can be attributed to the longer exposure time during printing which leads to a higher degree of incorporation of the drug within the polymeric matrix and stronger mechanical characteristics of these tablets, as previously shown. The results therefore suggest the influence of geometrical dimensions and mechanical characteristics on drug release profiles.

### 2.5. Differential Scanning Calorimetry (DSC)

**Figure 5:**
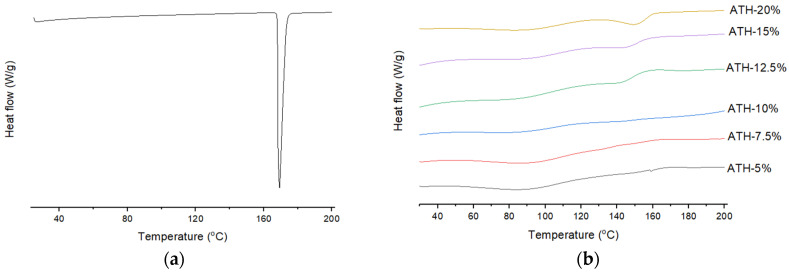
DSC (differential scanning calorimetry) thermograms of: (**a**) pure ATH prior to printing, and (**b**) the DLP 3D-printed tablets with different amounts of ATH.

DSC (differential scanning calorimetry) analysis of pure ATH and fabricated tablets was performed in order to investigate whether the drug was dispersed or dissolved within the polymeric matrix. The DSC thermogram of raw ATH exhibited a sharp endothermic peak at 169 °C due to melting of drug crystals. There is no sharp melting peak of ATH on the thermograms of powdered tablets, but broad endotherms between 140 and 160 °C can be observed during heating of formulations containing over 12.5% of ATH (Figure 5). It is difficult to distinguish these endotherms for formulations with a lower content of ATH. Although the absence of drug melting peaks indicates that the drug is dispersed in an amorphous form or dissolved in the polymer, dissolution of drug crystals during heating in DSC analysis cannot be excluded. Therefore, samples were further analyzed by XRPD (X-ray powder diffraction) and polarized light microscopy.

### 2.6. X-ray Powder Diffraction (XRPD)

**Figure 6:**
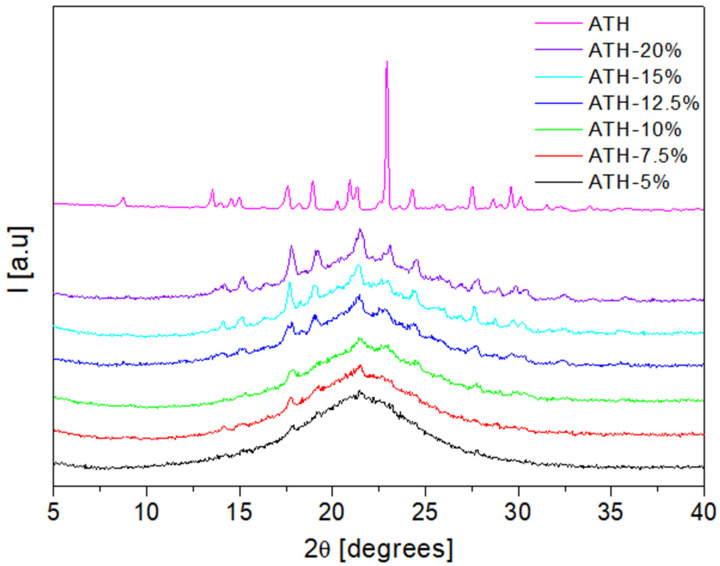
The X-ray powder diffraction (XRPD) patterns of raw unprocessed ATH and fabricated tablets.

XRPD was used to study the physical form of the active substance in the fabricated tablets. The X-ray diffractogram of pure ATH exhibited multiple diffraction peaks at 17.6°, 18.2°, 18.9°, 21.3°, 23°, 24.3° and 27.5° 2θ, which reflect its crystalline form (Figure 6). Diffraction peaks characteristic for crystalline ATH can be distinguished for formulations containing over 12.5% of the drug. The broadening of ATH peaks in formulations containing below 12.5% of ATH indicates the reduction of drug crystallinity. However, a peak positioned at 17.6 θ is present on the diffractograms of formulations with 5%, 7.5% and 10% of ATH, while a peak at 21.3 θ can be additionally observed on diffractograms of formulations with 7.5% and 10% of ATH. These results indicate that the drug is present in a crystalline form in all prepared formulations, with a particular reduction in drug crystallinity in formulations containing less than 12.5% of ATH. This is also confirmed with the microscopic observations, which are discussed in the following section.

### 2.7. Polarized Light Microscopy

Polarized light microscopy was used for crystal identification as well as for the observation of a cross section of the inner structure of fabricated tablets before and after the performance of dissolution testing.

**Figure 7:**
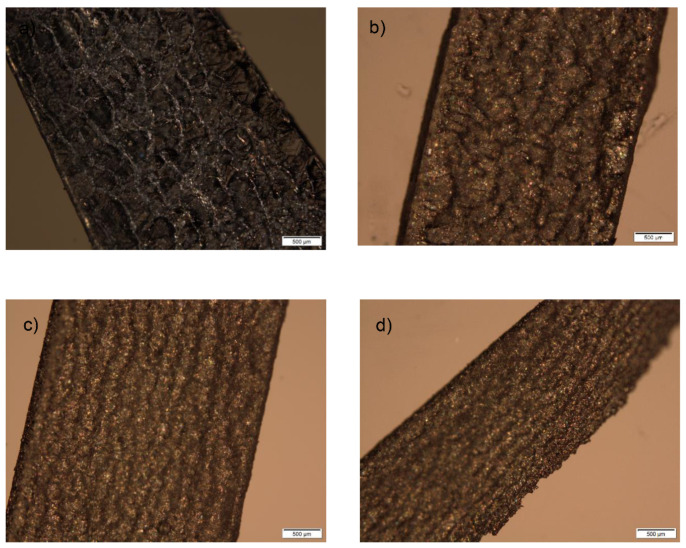
Cross section of tablets before dissolution testing containing: (**a**) 5%; (**b**) 10%; (**c**) 15%; (**d**) 20% of ATH.

As shown in Figure 7, crystals are present on the cross section of tablets prepared with different amounts of ATH (5–20%).

This provides additional evidence that ATH remains in tablets after printing in the crystalline state. Another significant observation is the uniform and homogenous distribution of the drug within the polymeric matrix, which further prevents the dose dumping phenomenon in which a relatively large amount of the drug in a controlled-release formulation is quickly released, resulting in a potentially toxic quantity of the drug being introduced into the systemic circulation. This is particularly dangerous in the case of potent drugs, which have a narrow therapeutic index (TI). 

**Figure 8:**
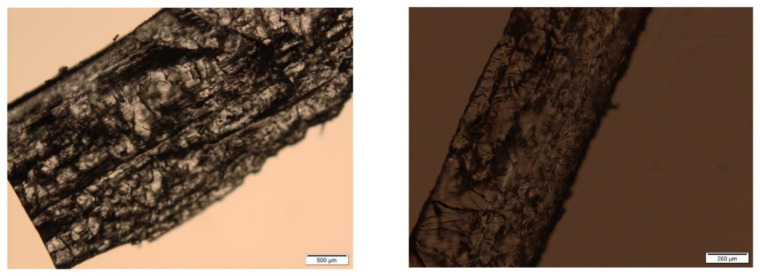
Cross section of tablets containing 5% of ATH after dissolution testing.

It was not possible to observe parallel layers in the printed tablets due to the presence of crystals of drug and the PI which covered them. On the other hand, on the photomicrographs of the cross section of the tablets after dissolution tests, layers are visible in some places, with the generation of cracks and channels which enabled the diffusion of the drug (Figure 8).

### 2.8. Fourier-Transform Infrared Spectroscopy (FTIR)

**Figure 9:**
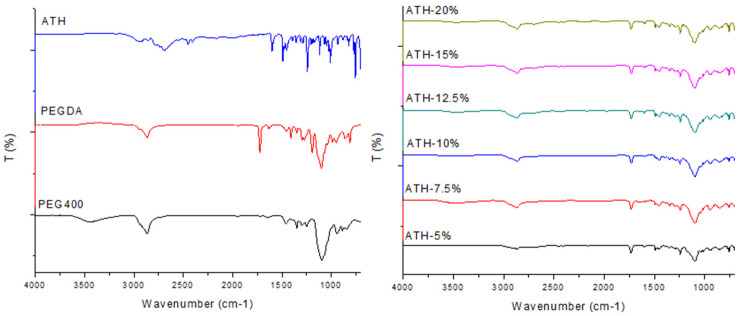
Fourier-transform infrared (FTIR) spectra of substances (**left**) and fabricated tablets (**right**).

Fourier-transform infrared (FTIR) spectroscopic analysis was used for the assessment of compatibility between the drug and polymers used for the preparation of 3D-printed tablets. The FTIR spectra for PEGDA, PEG 400, ATH powder and fabricated tablets are presented in Figure 9.

PEG 400 showed characteristic peaks at 3445 cm^−1^ (O-H stretching), 2866 cm^−1^ (C-H stretching) and 1096 cm^−1^ (C-O-C ether stretching) [41]. Peaks at 2866 cm^−1^ and 1096 cm^−1^ are also present in the spectra of the printed tablets. The spectrum of uncured PEGDA had characteristic peaks at 2866 cm^−1^ (CH3 stretching), 1721 cm^−1^ (C=O stretching) and 1636 cm^−1^ (acrylate C=C stretching) [27,42]. After the photopolymerization process, it was not possible to identify the acrylate-related peaks at 1636 cm^−1^, due to the conversion of C=C to C-C bonds [41]. The major peaks identified in the pure ATH spectra were clearly observed in the spectra of all fabricated tablets, which indicates the absence of interactions between drug and polymers. 

### 2.9. ANN Modeling

**Figure 10:**
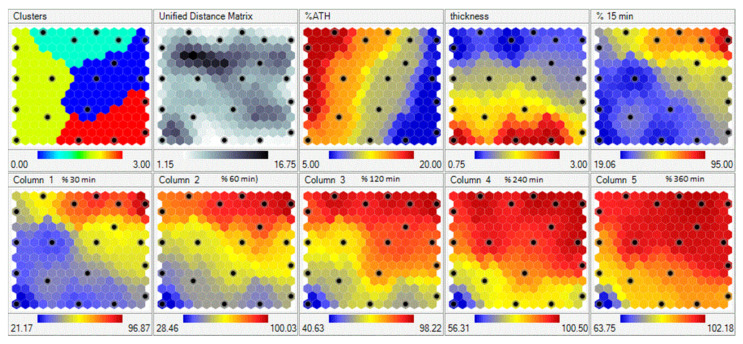
Self-organizing maps of drug release data; black dotes represent properties of the same nodes.

Data from 23 experiments were trained with the Kohonen learning rule in self-organizing maps (SOMs) with learning rate of 0.5. After training, SOMs are presented in Figure 10. Each input and output is presented as a separate region (map). The map consists of hexagonal cells, where each cell represents the SOM node. The node is associated with a data point to which it is closest. 

Data points (experimental runs) that are used for SOM training are presented as black dots in a map. Distribution of data through the region can be easily visualized on the maps, while the color scale of a variable gives us information about data clustering. For example, the black dot on the right top corner of the maps is the experimental run with the lowest percent of ATH (blue cluster), with tablet thickness of 0.75. For that experimental run, the drug release after 15 min was the fastest (red cluster in the top right corner of the map corresponding to drug release after 15 min). 

**Figure 11:**
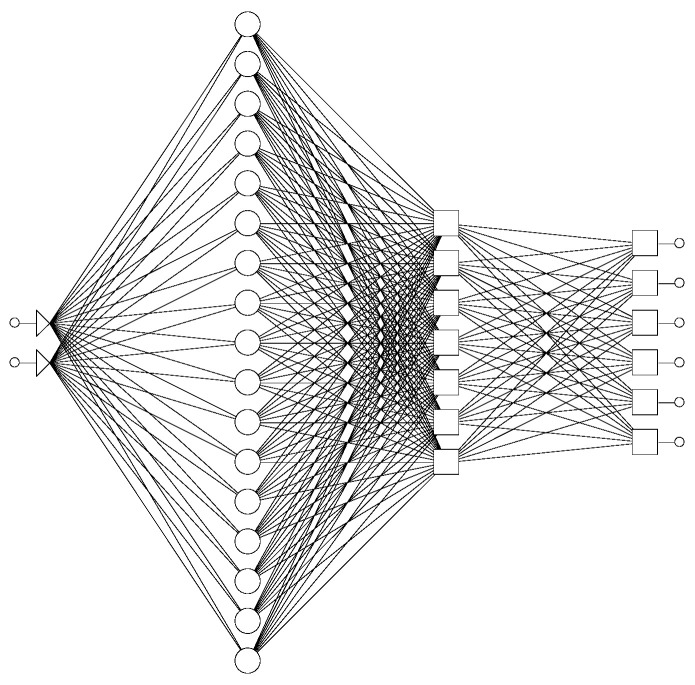
Schematic representation of the used GRNN (generalized regression neural network).

The next step in modeling was developing ANNs that could describe influence of tablet thickness and drug loading on ATH release from printed tablets. A total of 23 experimental runs were divided into training set (17), validation (4) and test set (2). A generalized regression neural network (GRNN) was trained (Figure 11). GRNNs have exactly four layers: input, a layer of radial centers, a layer of regression units, and output. The radial layer units represent the centers of clusters of known training data. This layer was trained by a K-means clustering algorithm, using smoothing factor 0.02. The maximal number of neurons in this layer is equal to number of training cases. During training, the number of units in the second layer was varied, and the lowest root mean square (RMS) for the training and validation data set was obtained with 17 units in the second layer. The third regression layer has one more unit then the output layer (7). The regression and output layer are trained extremely quickly using the specialized algorithm provided (called a division algorithm).

After training, values for RMS error were 0.126 for the training set, 0.040 for the validation set and 0.035 for the testing set. 

**Figure 12:**
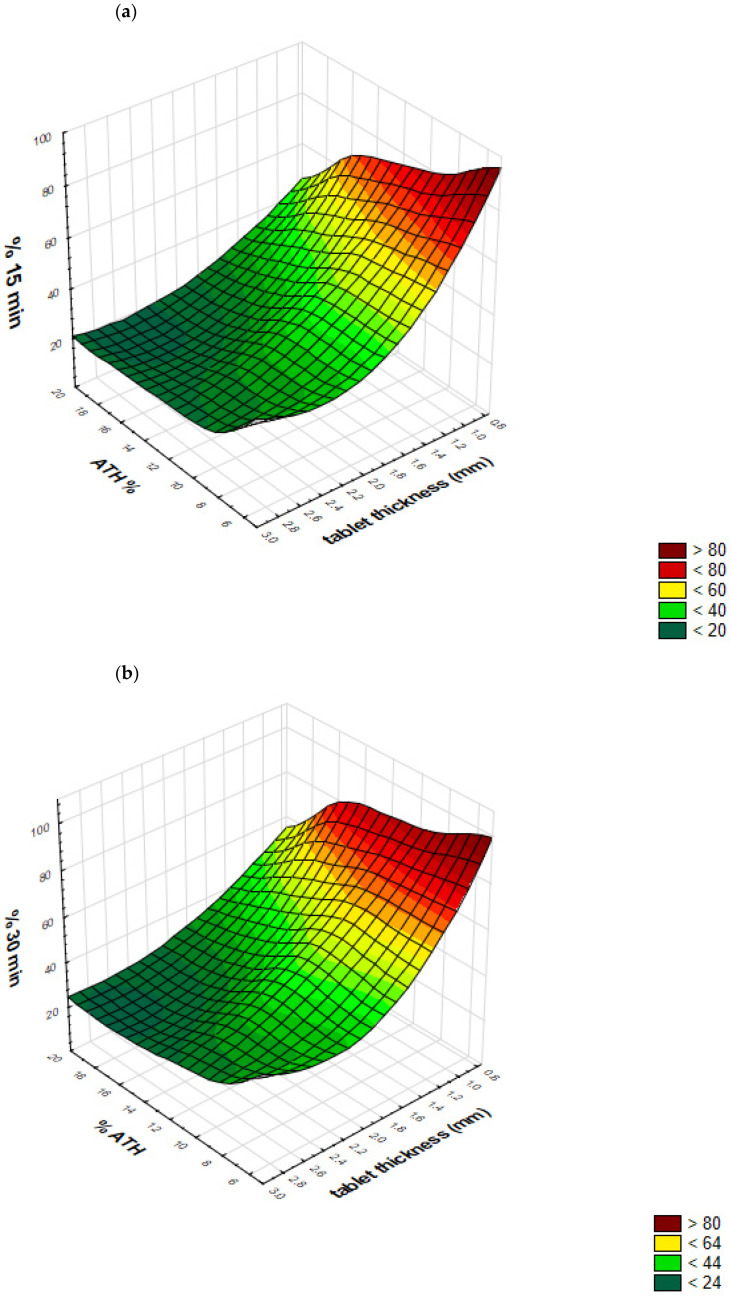

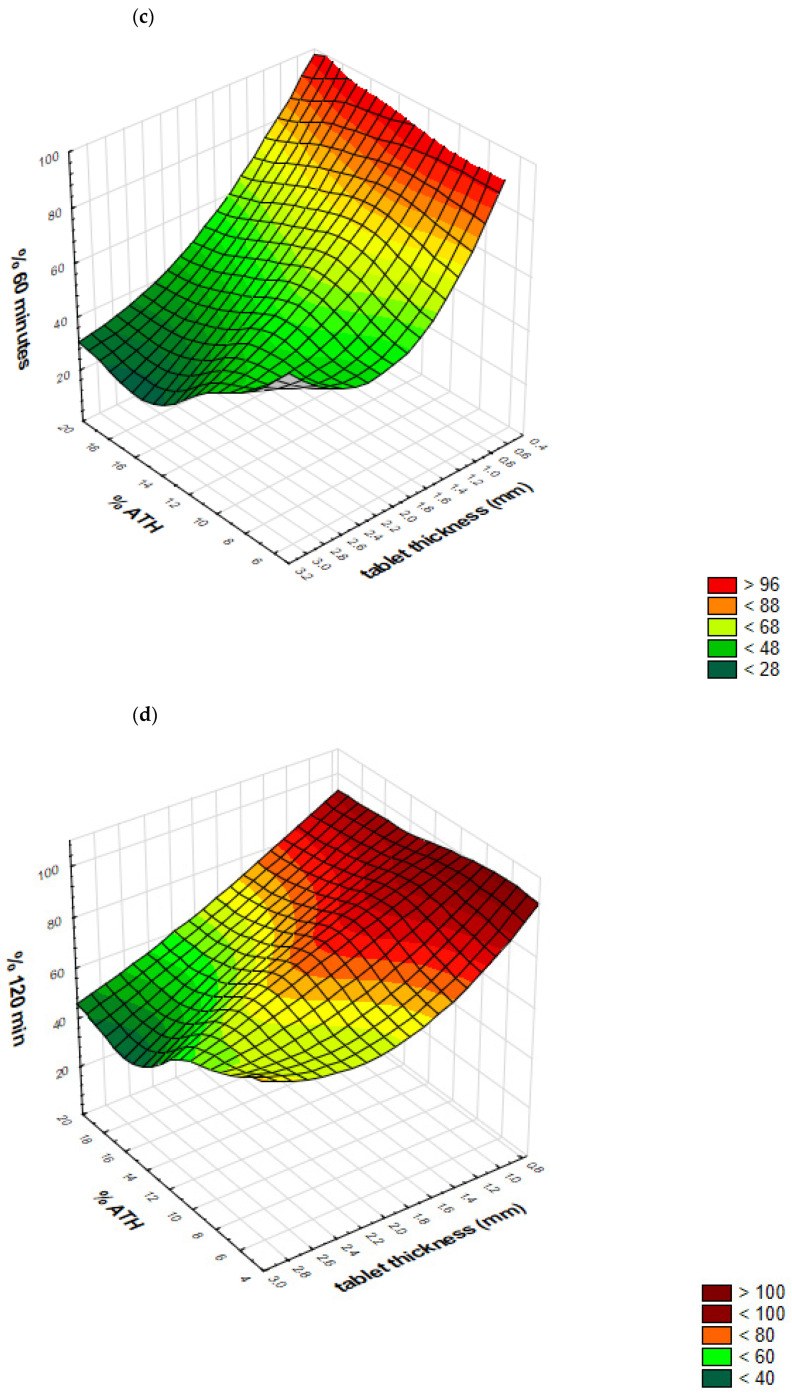

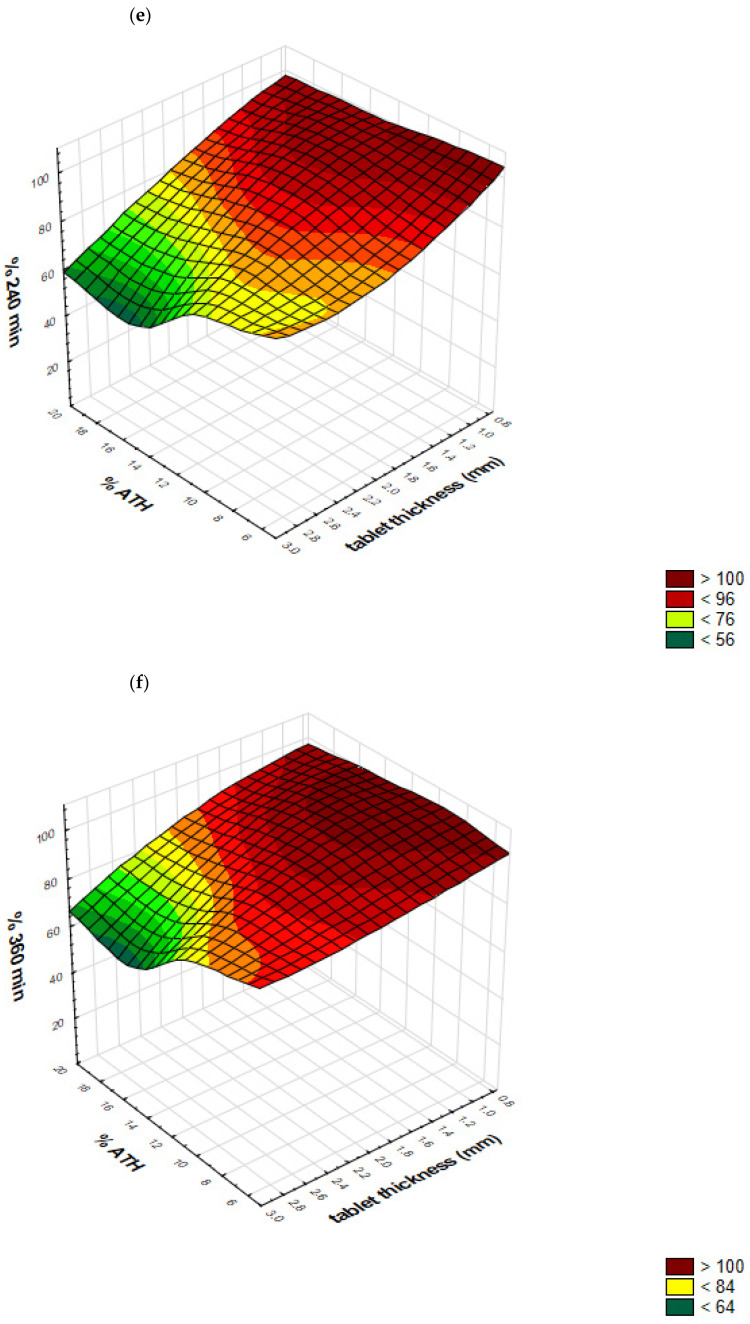
Response surfaces, representing outputs (Y1–Y6 percent of drug released after predetermined time intervals) as a function of drug loading (% ATH) and tablet thickness: (**a**) Y1—after 15 minutes; (**b**) Y2—after 30 minutes; (**c**) Y3—after 60 minutes; (**d**) Y4—after 120 minutes; (**e**) Y5—after 240 minutes; (**f**) Y6—after 360 minutes.

Response surfaces predicted by GRNN, representing outputs (Y1–Y6 percent of drug released after predetermined time intervals) as a function of tablet thickness and drug loading, are presented in Figure 12a–f. These response surfaces clearly show GRNN predictions of drug release, as a function of tablet thickness and drug loading.

**Figure 13:**
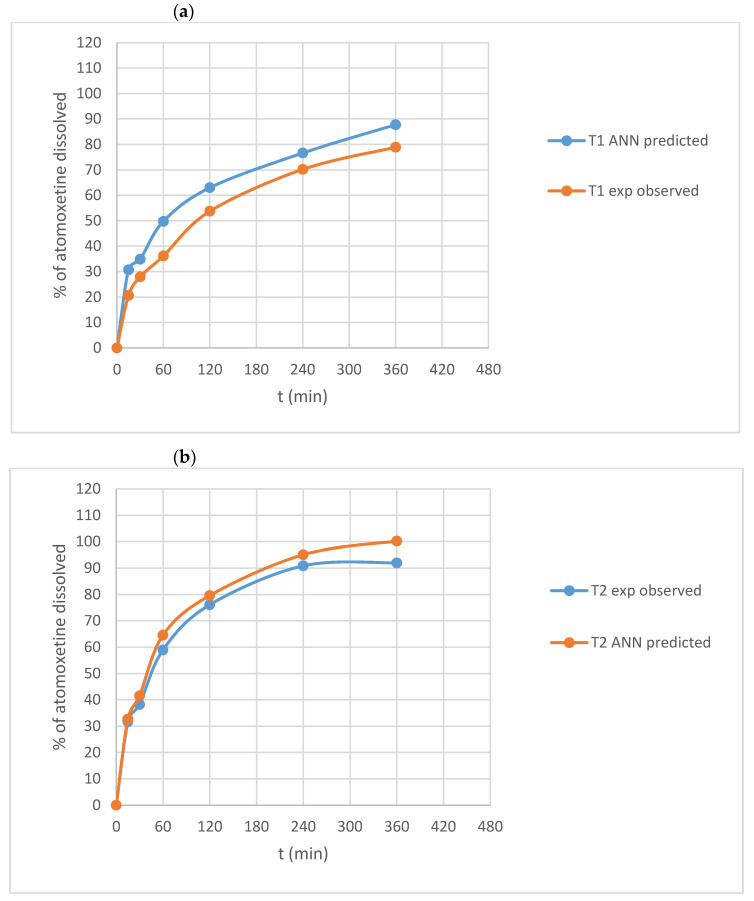
Experimentally observed versus GRNN-predicted dissolution profiles of ATH from formulations (**a**) T1 and (**b**) T2.

Additionally, for two test formulations, experimentally observed dissolution profiles versus profiles predicted using trained networks were evaluated using similarity factor *f*_2_. Dissolution profiles (experimentally observed vs. predicted) for T1 and T2 formulation are presented in Figure 13. Predicted dissolution profiles are similar to experimentally observed profiles, with calculated similarity factors of 51.05 and 70.13, respectively. 

These results demonstrated the ability of a trained GRNN to predict ATH dissolution profiles within a defined design space of tablet thickness between 0.75 and 3.00 mm and drug loading between 5% and 20%.

Additionally, within this design space, by the appropriate choice of tablet thickness and drug content, it is possible to achieve either immediate or prolonged release of ATH, using the same formulation and printing process.

## 3. Materials and Methods 

### 3.1. Materials

PEGDA, average MW 700, was obtained from Sigma-Aldrich, Tokyo, Japan. PEG 400, average MW 400, was purchased from Fagron B.V., Rotterdam, The Netherlands. ATH was kindly provided by Hemofarm AD, Vrsac, Serbia. DPPO was purchased from Sigma-Aldrich, Steinheim, Germany. All other chemicals used in the study were of analytical grade.

### 3.2. Methods

#### 3.2.1. Preparation of Photoreactive Mixtures

In this study, ATH was used as a model substance, PEGDA as the photopolymerizable monomer, DPPO as a photoinitiator and PEG 400 as an excipient which enhances incomplete drug release from tablets obtained by vat photopolymerization techniques. 

Content of ATH varied from 5.00% to 20.00% (*w*/*w*), while a 3:1 ratio of PEGDA/PEG 400 was the same in all formulations in order to obtain tablets with appropriate mechanical and release properties, based on a previously published study [30]. All formulations contained 10% water and 0.10% DPPO. Compositions of the formulations are given in Table 3. 

A total of 50 g of each formulation was prepared by mixing with the magnetic stirrer for 12 h until complete dissolution, keeping the mixture protected from light. The mixture was then transferred into the resin tray and printing was initiated. 

#### 3.2.2. 3D Printing of Dosage Forms

A cylindrical 3D model of the printed tablets was designed with Autodesk Fusion 360 software version 2.0.8809 (Autodesk Inc, San Rafael, CA, USA) and was exported as a stereolithography file (.stl) into the 3D printer software (Chitubox, version 1.7.0). All tablets were printed with Wanhao Duplicator 8 printer (Wanhao, Zhejiang, China). The diameter of all tablets was the same (8 mm) but their thickness was varied in the way shown in Table 4.

In order to optimize the printing process, preliminary experiments were conducted to establish printing parameters which will enable rapid and successful printing. In accordance with the results of these experiments, all tablets were printed with the same parameters shown in Table 5. 

#### 3.2.3. Mass and Dimension Variation

3D-printed tablets were washed with 2-propanol and wiped up with filter paper immediately after printing, to remove any excess uncured formulation on the surface of the tablets. Tablets were then weighed on an analytical balance (Kern & Sohn, Germany) and measured (diameter and thickness) using a digital caliper (Vogel Germany GmbH & Co. KG, Kevelaer, Germany). Measurements were performed on 10 tablets for each formulation. 

#### 3.2.4. Mechanical Properties of 3DP Tablets

It was not possible to perform a conventional hardness test as the tester could not break some of the fabricated tablets, particularly the thinnest one, due to their movement and deformation into the tester machine. Furthermore, because of the smoothness and elasticity of fabricated tablets, the results of a conventional friability test would not provide any significant information. An alternative approach, based on the measurement of the force required to push a needle probe into a tablet to a particular depth, was used to determine the effect of the drug load on mechanical characteristics of tablets. Testing was performed on an EZ-LX Compact Table-Top Testing Machine (Shimadzu, Japan). The test was performed on 1-mm-, 2-mm- and 3-mm-thick tablets, for which the penetration depth was set at 0.5 mm, 1 mm and 2 mm, respectively. The speed of the needle probe was 2 mm/min and three tablets of each formulation were examined. 

#### 3.2.5. Determination of Drug Content in 3DP Tablets

The drug content in 3D-printed tablets was determined by UV/VIS spectrophotometry (Evolution 300, Thermo Fisher Scientific, Waltham, MA, USA) at the wavelength of 270 nm [43]. For standard preparation, 10 mg of ATH was dissolved in 100 mL of distilled water, shaken in an ultrasonic bath Bandelin-Sonorex RK102H (Sonorex-Bandelin, Berlin, Germany) for 15 min at room temperature, cooled and then filtered through 0.45 µm filters (Millipore, Bedford, MA, USA). For test preparation, three tablets of each formulation were crushed using a mortar and pestle. The mass of each tablet equivalent to 10 mg of ATH was weighed and dissolved in a volumetric flask with 100 mL of distilled water and shaken in an ultrasonic bath for 15 min. Samples then underwent the same procedure as described for standard preparation. 

#### 3.2.6. In Vitro Drug Release Testing

Drug dissolution profiles of the printed tablets were obtained by USPII Erweka DT 600 (Erweka, Langen, Germany) apparatus. Tablets were placed in 500 mL of distilled water for 8 h. The paddle speed was fixed at 50 rpm, and the testing was conducted at 37 ± 0.5 °C. Samples (5 mL) were withdrawn at 15, 30, 45, 60, 120, 180, 240, 300, 360, 420 and 480 min time intervals, filtered through 0.45 µm filters (Millipore, Bedford, MA, USA) and the amount of released ATH was determined UV spectrophotometrically at 270 nm (Evolution 300, Thermo Fisher Scientific, Waltham, MA, USA). According to Krkobabic et al., the type of apparatus (paddle apparatus or flow-through cell) and medium (distilled water or 0.1 M HCl) do not significantly affect the ATH dissolution rate from 3D-printed tablets [30].

#### 3.2.7. Differential Scanning Calorimetry (DSC)

DSC was performed on a DSC 1 instrument (Mettler Toledo, Giessen, Germany). Precisely weighed 5–10 mg of pure ATH and powdered tablets were placed in pierced aluminum pans and subjected to heating at 10 °C/min in the range from 25 to 200 °C under constant nitrogen gas flow of 50 mL/min. An empty aluminum pan was used as a reference. The obtained data were further analyzed in the STARe software (version 12.10, Mettler, Toledo). 

#### 3.2.8. X-ray Powder Diffraction (XRPD)

XRPD measurements were done on a Philips PW 1050 powder diffractometer at room temperature with Ni-filtered Cu Kα 1,2 radiation, scintillation detector and Bragg–Brentano focusing geometry. The intensity and voltage applied were 40 kV and 30 mA. The XRPD patterns were taken within the angular range of 5–40° 2θ in steps of 0.05° and scanning time of 4 s per step. 

#### 3.2.9. Polarized Light Microscopy

An Olympus BX53-P polarized microscope (Olympus, Tokyo, Japan) with UPLFLN4XP and UPLFLN10XP objectives was used for visual examination of a cross section of the tablets’ internal structure before and after dissolution testing, as well as for crystal detection. Photos were acquired using cellSens Entry Version 1.14 software (Olympus, Tokyo, Japan). 

#### 3.2.10. Fourier-Transform Infrared Spectroscopy (FTIR)

Interactions between the drug and polymers were assessed using FTIR spectroscopy. Spectroscopic analyses of raw materials and crushed tablets were performed using a Nicolet iS10 (Thermo Scientific, Waltham, MA, USA) FTIR spectrometer, equipped with a single reflection ATR system (Smart iTR, Thermo Scientific, Waltham, MA, USA) with diamond plate and ZnSe lens. The spectra were collected in the frequency range from 4000 to 650 cm^−1^, with the resolution of 2 cm^−1^.

### 3.3. ANN Modeling

For ANN modeling, two types of neural networks were used. Firstly, a self-organizing map (SOM), as an unsupervised artificial neural network, was used in order to help us to visualize the influence of inputs (tablet thickness and drug loading) on ATH release from printed tables. Peltarion^®^ software (Synapse, Sweden) was applied for SOM development and visualization. 

Secondly, a generalized regression neural network (GRNN) was used for model development that could predict drug release from printed tablets. This network was built using TIBCO Statistica^®^ Software 13.5.0 (StatSoft Inc).

For both networks, input variables were: X1, tablet thickness (from 0.75–3.00 mm) and X2, ATH loading (from 5% to 20%). Dependent variables (outputs) were: percent of drug release after 15 min (Y1), 30 min (Y2), 60 min (Y3), 120 min (Y4), 240 min (Y5) and 360 min (Y6).

Data from 23 experimental runs were divided in training, validation and test sets (17:4:2). The best network was chosen based on the least RMS values for training, validation and test data set.

## 4. Conclusions

In this study, the possibility of successful fabrication of tablets with immediate- and modified-release characteristics containing different drug loadings with DLP 3D printing has been demonstrated. This further emphasizes the potential of this technology in the production of more acceptable dosage forms containing doses that are adjusted to the patient’s characteristics, which is particularly important for pediatric and geriatric populations, resulting in favorable therapy outcomes and adherence, and reduction of adverse effects and drugs toxicity. 

## Figures and Tables

**Table 1:** Measured weight, diameter, thickness and drug content (mean ± SD).

Formulation	Weight (mg)	Diameter (mm)	Thickness (mm)	Drug Content (mg)
D5-1.00	35.01 ± 2.90	7.88 ± 0.22	0.92 ± 0.10	2.06 ± 0.04
D5-1.25	54.30 ± 8.80	7.97 ± 0.07	1.25 ± 0.04	2.44 ± 0.05
D5-1.50	70.50 ± 5.40	8.02 ± 0.04	1.48 ± 0.03	3.14 ± 0.01
D5-1.75	84.93 ± 6.50	8.05 ± 0.06	1.75 ± 0.02	4.15 ± 0.20
D5-2.00	110.38 ± 14.80	8.22 ± 0.38	1.90 ± 0.20	4.62 ± 0.15
D5-3.00	215.80 ± 15.60	8.62 ± 0.32	2.93 ± 0.09	7.76 ±0.20
D7.50-2.50	116.22 ± 6.10	8.14 ± 0.21	2.43 ± 0.04	7.16 ± 0.02
D10-1.00	45.12 ± 2.00	7.96 ± 0.11	0.98 ± 0.03	3.71 ± 0.03
D10-1.25	65.57 ± 8.03	8.03 ± 0.04	1.25 ± 0.06	5.06 ± 0.05
D10-1.50	85.97 ± 10.20	8.10 ± 0.09	1.50 ± 0.02	6.41 ± 0.09
D10-2.00	94.35 ± 5.80	8.08 ± 0.10	1.96 ± 0.08	7.18 ± 0.06
D10-3.00	188.15 ± 15.6	8.21 ± 0.12	2.92 ± 0.15	12.84 ± 0.50
D12.50-1.50	78.89 ± 7.20	8.29 ± 0.24	1.44 ±0.06	8.47 ± 0.40
D15-0.75	40.11 ± 6.40	8.04 ± 0.07	0.77 ± 0.03	5.20 ± 0.03
D15-1.00	49.89 ± 8.10	8.11 ± 0.11	1.02 ±0.09	6.37 ± 0.03
D15-1.50	95.39 ± 9.20	8.33 ± 0.17	1.48 ± 0.04	11.72 ± 0.03
D15-2.00	111.84 ± 14.60	8.30 ± 0.19	1.96 ± 0.10	15.15 ± 0.80
D15-3.00	207.53 ± 29.20	8.87 ± 0.31	2.95 ± 0.08	33.16 ± 0.75
D20-0.75	48.10 ± 5.40	8.22 ± 0.17	0.78 ± 0.03	8.10 ± 0.50
D20-1.00	54.29 ± 5.10	8.02 ± 0.06	1.01 ± 0.04	9.05 ± 0.74
D20-1.50	110.14 ± 6.80	8.47 ± 0.09	1.51 ± 0.06	18.14 ± 0.23
D20-2.00	118.03 ± 10.60	8.40 ± 0.33	1.95 ± 0.08	21.20 ± 0.58
D20-3.00	214.35 ± 18.20	9.02 ± 0.26	2.94 ± 0.09	37.48 ± 1.41

**Table 2:** Measured force in the test with needle probe (mean ± SD).

Amount of ATH	Tablet Thickness	Force (N)
5%	1.00	1.49 ± 0.05
	2.00	2.98 ± 0.12
	3.00	6.00 ± 0.17
10%	1.00	1.81 ± 0.07
	2.00	3.35 ± 0.22
	3.00	7.40 ± 0.09
15%	1.00	2.27 ± 0.01
	2.00	5.58 ± 0.05
	3.00	11.82 ± 0.25
20%	1.00	3.26 ± 0.03
	2.00	7.14 ± 0.08
	3.00	13.55 ± 0.30

**Table 3:** Compositions (*w*/*w*) of photoreactive mixtures used for 3D printing.

Formulation	ATH	PEGDA	PEG 400	Water	DPPO
D5	5.00	63.70	21.20	10.00	0.10
D7.5	7.50	61.80	20.60	10.00	0.10
D10	10.00	59.93	19.97	10.00	0.10
D12.5	12.50	58.05	19.35	10.00	0.10
D15	15.00	56.18	18.72	10.00	0.10
D20	20.00	52.43	17.47	10.00	0.10

**Table 4:** Dimensions (diameter and thickness) of created 3D models.

Formulation	Thickness (mm)	Diameter (mm)
D5	1.00, 1.25, 1.50, 1.75, 2.00, 3.00	8.00
D7.5	2.50	8.00
D10	1.00, 1.25, 1.50, 2.00, 3.00	8.00
D12.5	1.50	8.00
D15	0.75, 1.00, 1.50, 2.00, 3.00	8.00
D20	0.75, 1.00, 1.50, 2.00, 3.00	8.00

**Table 5:** Parameters of printing process.

Parameter	Value
Layer height	0.1 mm
Bottom layers	5
Exposure time	20 s
Bottom exposure time	30 s
Bottom lift distance	5 mm
Lifting distance	5 mm
Bottom lift speed	300 mm/min
Lifting speed	300 mm/min
Retract speed	300 mm/min

## Sample Availability

Samples of the compounds are not available from the authors.
